# Halcyon Days*From Dr. Daniel’s, Recollections of a Rebel Surgeon.

**Published:** 1910-05

**Authors:** 


### Abstracts and Selections.

### Halcyon Days.*

I see by the papers, said our Genial Visitor, that today is Commencement Day at the Texas Medical College. Dan'els, do you ever think of the time when you got your sheepskin? To me it was one of the most trying ordeals of my life, except, perhaps, that

*From Dr. Daniel's, Recollections of a Rebel Surgeon.

time when the Yankees killed me, and I reckon it's the same with most boys. "In the spring the young man's fancy lightly turns to thoughts of love," says Tennyson; but the average medical student crams on Smith's Compend, and prepares for examination. With hesitation, trepidation and perspiration, he approaches that green baize door which, veiling his future, conceals a terror in the shape of a bald-headed professor, in whose hands hangs the destiny of many fellers, each not by a thread but by a string—of hard questions. "Happy they, the happiest of their kind," to whom Pat, the janitor, hands a long round tin box next day, while with a grin he suggestively protrudes his left hand for the expected fee, never less than a V.

Who so proud, then, as they, the fledglings, the new-born medicos? as when next they meet, the old familiar "Tom" and "Harry" are dropped, and it's "Good morning, *Doctor;* accept my congrats. Didn't old Blimber make a fellow sweat?"

"Oh, pshaw, *Doctor,* he was *nothing* to old Bones when he got me on the ligaments. I was up-to-date, tho', you bet; *crammed.* So long, *Doctor.*"

(Another two):

"Ah, good morning, Doctor; got through, I hear. Yes, it *was* tough. Be on hand tonight, of course, with your swallow-tail." (Exit.)

The palpitating part of it had only *begun,* however, in the green-room. (How provokingly old Bones *did* grin when he asked them to "give him the ligaments of the neck.") All those young M. D.'s have to stand the battery of bright eyes tonight at the opera house; and in that large and fashionable audience, all a-flutter with fans and furbelows, every young feller has a bright particular pair of eyes that to him look like the rising sun, as he steps out in response to his name to get his sheepskin; while to the owner of said pair of rising-sun orbs, that particular name on the program, it may even be "Grubs," blazes with a holy light, quite eclipsing all the others. (And the band played Annie Laurie.)

Then, the first time *she* calls Harry "Doctor"—oh, not for the crown of an Indian prince would he exchange that proud title. (We've been there, tho' it was in the long, long ago, memory brings back the days that are no more.)

And at the ball; and after the ball; what "medicine" (heart-excitants mostly, I fear) is talked, as arm in arm each happy couple promenades beneath the vine-clad trellis, or—drop the curtain here; the "sweetness" of that "faithful watch-dog's honest bark," that Byron tells us about, "baying deep-mouthed welcome," as in after years we "draw near home"—any rainy dark night after a ten-mile ride for a bare "thankee," is just only brown sugar to double distilled saccharine, compared to the bliss of those moments spent with Dulcinea the first evening he wore his title and his pigeon-tailed coat; as they told and listened 'neath the umbrageous shades of those grand old oaks, to the old, old tale; it is always the same; told with variations often, perhaps, but always the same old tale—and ever new; told with the eyes, for "the heart doth speak when the lips move not"—so that when flashed from a woman's eyes even a savage can comprehend "two souls with but a single thought," etc. Ah me; would I were a boy again—or rather a young doctor sprouting his first mustache. How much medicine we did know as that time, good gracious! "The wonder grew," sure enough with me, that "one small head could carry" it.

Now, I'm going to tell you a joke about that same head. I haven't *got* a small head; I've got a big head.

About six years subsequent to the events I'm telling about (that is, the occasion on which I received my diploma), I was myself a professor, and had to ask the boys hard questions; I was "Old Bones" myself. One day coming out of the hospital where I had just been lecturing—I had on a new spring style hat. One of the students admired it and asked to look at it. I took it off and handed it to him. He tried it on and it come down over his ears. The boys laughed at him and he remarked:

"Doctor, you have a very large head."

I said: "Yes, larger than the average I believe."

One young scamp looked roguishly out the corners of his eyes at me and said slyly:

"It's a little swelled, ain't it, Doctor?"

Well, yes; I believe now that it *was* swelled. I can look back at that period of my life—in fact at most of it, and realize what a fool I was. I do think now that it was a mercy that the fool-killer never got me, and sometimes I think it's a pity he didn't.

But I've digressed. I was saying that in our young days we are very conceited and think we know a great deal of medicine. It takes an average lifetime to find out that we don't know anything worth mentioning, as Dickens said of Mr. Bailey's nose; he had none "worth speaking of." Somehow one's head seems to leak medical knowledge as the bones harden and the sutures close up. Just the reverse of what we would expect, but it is a fact. I think most doctors of my age will admit it—the older we get the less we know. Crowded out, p'raps, to make room for a recollection of our uncollected bills (or unpaid ones), or by family cares and calculations how we are to make a $2 fee buy shoes and stockings for the baby, and a new bonnet for the dear wife—her of the sunrise eyes of long ago.

Ah yes, spring time *is* "commencement" time; and the output of the new issue of—I like to have said "greenbacks," or "government bonds," so absorbed was I in studying out the above financial sphynx—the output of the new generation of doctors is large. I have not kept a memorandum of the total; each college is making them by the score, out of raw material (very raw, some of it), that beyond a doubt will make the future Sir Andrew Clark, the S. D. Gross, the Austin Flint and the Marion Sims of the next generation.

To them all, to those who are properly imbued with the love of science, who have chosen medicine not as a money-getter alone, I say—*"aim high."* What was possible to the poor Southern boy, Sims, Wyeth, Nott; or to the lamented Quimby, or Jno. B. Hamilton—a farmer's boy—is possible to you. Do not put away your books now that you have your diploma; you have only *graduated*—you have not finished—you have only begun, prepared yourself to study and learn. Today is truly your "commencement" day. "Drink deep, or touch not the Pierian spring." Let not alone the sunrise eyes of your beloved inspire you; determine to win for her a place where in after years she may not be ashamed of her young doctor. "The hill whereon Fame's proud temple shines afar" *is* hard to climb; but it has been climbed. What others can do, you can do; so my dear boys—I *beg* your pardon—dear young doctors—aim high!

But after the new has rubbed off, after a life of toil, too often thankless, most often unremunerative, things look a little different to the doctor, don't they, Dan'els? You know; you've been through the mill; so've I.

Now, by contrast (I've just given you fellers a glimpse of the panorama as she spread out at the start), I'll give you a picture drawn later in life. I'm reminded of it by the foregoing reminiscences of commencement day. This thing I'm a giving you now—here, Hudson, read this—was written by yours truly for a young lady whom I thought a heap of, one time. She jokingly said that doctors "put on" a good deal; that it was all stuff about their having a hard time, etc. Just for fun I wrote this for her and my wife got hold of it, and like everything else I ever wrote *she,* kind, trusting soul, thought it was "smart." (Hudson reads):

### THE DOCTOR'S LAMENT.

### (TO HIS LADY LOVE.)

That's what I called it, said the Old Doctor, before Hudson began to read, but it might appropriately be called "Days that weren't quite so halcyon"—eh, Dan'els? (Hudson reads):

"Your life leads down by peaceful, tran quil rivers
Whose shady bank the cooling breeze invites;
While mine—alas! is spent 'midst torpid livers,
And similar sad and melancholy sights.

To you the perfumed air is rich with sounds
As sweet as when first Sappho's harp was strung;
While I in sun and dust must take my weary rounds
To feel a pulse or view a coated tongue.

The choicest books beguile your leisure hours,
And sooth to sleep, or wake to sympathetic tears;
But woe is me, I spend my feeble powers
'Midst fever's fervid heat, or checking diarrheas.

You sleep in peace on soft and downy beds,
And dream, perhaps, of flowers in sunlit lands;
While I, no doubt, am soothing aching heads,
Or humbly giving aid by pulling hands.

Your lovers kneel before you in rapturous adoration,
And tales of love in mellifluous measures pour;
Creditors besiege me—they are my abomination,
And moneyless patients daily throng my office door.

Thy gentle pen, anon, the choicest thoughts indite,
That dwell within thy gentle breast, or tender mem'ry fosters;
Prescriptions I, with stubby pencil write:—
'Recipe: *misce et fiat haustus.*'

Alas! alas! my lady love! I tire indeed of these
Old scaly scalps of seborrhea and eczematous hands;
Let's trim our sails to catch an outward breeze,
And *endosmose* in pleasant foreign lands—

Away beyond the seas, on some peaceful, starlit isle,
Where rhythmic wavelets break on coral strands;
There, there'll be no fever, pus nor bile,
And a'down the happy years we'll pull each other's hands."

---

Judgment must be used in employing the iodides to diagnose syphilis as many other conditions are improved by this treatment, notably actinomycosis, chronic rheumatoid deposits and chronic lymphadenitis.—*American Journal of Surgery.*

